# Cumulative Early‐Life Adversities Predict Short‐ and Long‐Term Survival in a *Critically Endangered* Lemur ( *Propithecus verreauxi* )

**DOI:** 10.1002/ece3.74194

**Published:** 2026-08-14

**Authors:** Claudia Fichtel, Peter M. Kappeler

**Affiliations:** ^1^ Behavioral Ecology & Sociobiology Unit German Primate Center, Leibniz Institute for Primate Research Göttingen Germany; ^2^ Department of Sociobiology/Anthropology, Johann‐Friedrich‐Blumenbach Institute of Zoology and Anthropology University Göttingen Göttingen Germany; ^3^ Mention Zoologie et Biodiversité Animale Université d'Antananarivo Antananarivo Madagascar

**Keywords:** early‐life adversity, fitness, life history, mortality, primates, *Propithecus*, survival

## Abstract

Adversities in early life compromise fitness components later in life, but the cumulative effects of multiple adversities and their timing across the lifespan remain poorly understood. To contribute comparative data from an independent primate radiation, we leverage long‐term demographic data from a wild lemur population to examine the effects of maternal loss, presence of an older sibling, maternal age, group size, temperature and rainfall on survival of 242 infants across the lifespan. We found that differently operationalized cumulative indices of early adversities predicted infant survival but did not predict survival better than single predictor models. Infant survival was reduced by maternal loss, the absence of an older sibling and large group size at death. Survival to adulthood and female longevity were positively associated with higher rainfall during infancy and negatively by larger group sizes at death; infant survival to adulthood also declined with increasing maternal age. Cumulative adversities early in life had no persistent negative long‐term effects on survival, but sample sizes decreased in the older age classes. However, rainfall as a proxy for food availability in early life had long‐term effects, with higher rainfall increasing the odds of survival to adulthood and female longevity. Hence, social and demographic adversities had stronger effects than environmental ones for early infant survival, but environmental effects in early life also appear to be important drivers of demography and life history in these critically endangered primates.

## Graphical abstract

Infant survival of wild Verreaux's sifakas was impacted by different cumulative indices of early adversities. Of the single factors, maternal loss, large group size and the absence of an older sibling decreased the odds of survival across the lifespan. Adversities early in life have persistent negative long‐term effects on survival.

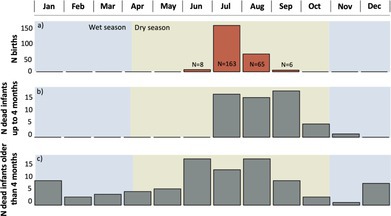

## 1 Introduction

If adverse environmental and social conditions do not have instantaneous lethal effects (e.g., Foley et al. 2008; Walker et al. 2023), life‐history theory predicts that individuals experiencing adversity during early development may face lifelong negative fitness consequences if the corresponding allostatic load cannot be effectively compensated (Lindström 1999; Lu et al. 2019; Nettle and Bateson 2015). These plastic developmental effects are proximately mediated by compromised growth and immunocompetence, stress responsiveness and sexual attractivity, for example (Eyck et al. 2019; Gustafsson et al. 1995; Lu et al. 2019). If environmental adversities affect entire cohorts, these consequences may also impact dynamics at the population level (Desforges et al. 2021; Sæther 1997) and, if the effects are persistent, ultimately life‐history evolution (Beckerman et al. 2002). Even though animals are exposed to a myriad of social, biotic and abiotic factors that act as stressors between fertilization and death, the potential cumulative impact of multiple stressors, particularly early in life, remains poorly known (Orr et al. 2020; Ortiz‐Ross and Blumstein 2024; Pirotta et al. 2022). Studies of short‐ and long‐term fitness effects of multiple simultaneous stressors are therefore of fundamental interest to scholars of ecology and evolution as well as of practical importance in conservation biology in the context of global change biology.

Adversities, defined as stressors that can move individuals out of their normal operating range (Pirotta et al. 2022) or disruptions of homeostasis (Dettmer and Chusyd 2023), can impact individuals on several time scales. Because the developing embryo interacts with the environment soon after fertilization (Bateson 2017), parental effects and environmental stressors may impact developmental trajectories already prenatally (Berghänel et al. 2016; Schülke et al. 2019). Early life adversities experienced postnatally often have additional rippling developmental effects on health and survival (Lu et al. 2019; Nettle and Bateson 2015; Rosenbaum et al. 2025). An individual's health, survival and fecundity may even be affected by early adversities experienced by its parents during their early development (Yin et al. 2019; Zipple et al. 2019). These events and processes have long been recognized to also play an important role in shaping human health (Bateson et al. 2004; Lummaa and Clutton‐Brock 2002), and have therefore been studied most intensively in laboratory animals (McGowan and Matthews 2018), but an increasing number of studies of adversity‐related fitness consequences have also been conducted in wildlife (Dettmer and Chusyd 2023).

Despite an increasing number of relevant studies, generalizations about fitness consequences of early‐life adversities (ELAs) in wild animals remain difficult because previous studies focused on different fitness proxies as well as on various types and numbers of adversities (Dettmer and Chusyd 2023; Morrison et al. 2023). Moreover, these studies varied in whether and how they analyzed cumulative effects of multiple stressors (i.e., as individual predictors, cumulative ELA categories or a single cumulative index; Gicquel et al. 2022; Morrison et al. 2023; Ortiz‐Ross and Blumstein 2024; Petrullo et al. 2024; Tung et al. 2016). Because ELAs are not mutually exclusive, it is increasingly recognized that their cumulative effects should be assessed, but the different conceptual and methodological approaches used so far have yielded divergent results.

Some early studies explored the fitness consequences of experimental manipulations of a single ELA, for example the consequences of brood size manipulations for physical development and mortality in captive zebra finches ( *Taeniopygia guttata* ; De Kogel 1997). Similarly, some field studies reported the effects of single natural adversities on fitness, such as the increased mortality of young male African elephants ( *Loxodonta africana* ) after a drought (Foley et al. 2008) or survival consequences of severe winter storms on several species of seabird (Laurenson et al. 2025).

Studies of the effects of two or more stressors on fitness components provided more comprehensive insights about the complex relationships among multiple stressors and various fitness proxies (Orr et al. 2020; Pirotta et al. 2022). In wild roe deer ( *Capreolus capreolus* ), for example, harsh early‐life conditions mediated by habitat quality and annual food availability imposed cumulative developmental constraints with major consequences for reproduction and survival (Douhard et al. 2014). An influential study of savannah baboons ( *Papio cynocephalus* ) revealed that cumulative survival consequences of experiencing three or more of ELAs resulted in an impressive reduction in longevity of about 10 years (Tung et al. 2016). Similar cumulative effects were also reported for spotted hyenas ( *Crocuta crocuta* ; Gicquel et al. 2022; Strauss et al. 2020), but in rhesus macaques ( *Macaca mulatta* ), negative cumulative effects were documented for both sexes in one study (Patterson et al. 2024), but limited to adult male survival in another study (Gonzalez et al. 2023), whereas in gorillas ( *Gorilla gorilla* ) adult males experiencing 3 or more ELAs actually enjoyed higher survival (Morrison et al. 2023). Thus, the effects of ELAs appear to be variable, and the fact that different studies used different developmental milestones for evaluating their effects has further added to the challenges of deriving general insights. While no single additional study will resolve these discrepancies, more studies are indicated to broaden the taxonomic and ecological diversity of study systems to contribute comparative data on this important topic.

Here, we report on the effects of multiple ELAs on survival in Verreaux's sifakas ( *Propithecus verreauxi* ), group‐living arboreal primates endemic to southwestern Madagascar, where they live in groups of about six individuals (Kappeler and Fichtel 2012). They can live up to 25 years (median longevity: 12 years) and can produce a single infant every year (mean interbirth interval: 1.25 years) from their fourth year of life (mean age of first reproduction: 5.6 years) onwards without any sign of reproductive senescence (Kappeler, Pethig, et al. 2022). Infant mortality is high (see below), and their physical development is much faster than expected for a primate of their size (Malalaharivony et al. 2021). A study in Beza Mahafaly Reserve revealed that impending maternal death, a proxy of maternal condition, predicted higher offspring mortality (Zipple et al. 2021), but other potential ELAs driving infant mortality in sifakas have not been studied. However, a previous study indicated that large group size compromises sifaka survival generally (Kappeler and Fichtel 2024), highlighting a potential role for intraspecific competition already in early life. As these sifakas are *Critically Endangered* (Louis et al. 2020), understanding the relative importance of potential drivers of mortality and their potentially cumulative effects can not only provide insights into the life history evolution of a member of a primate radiation that evolved independently of the anthropoid primates studied so far (Kappeler 1997), but it would also help to identify stressors that may ultimately impact their survival as a species (Brandon et al. 2024).

Because these primates evolved in isolation from other primates on Madagascar for about 50 million years (Everson et al. 2025), our study offers an independent test of the hypothesis that the effects of multiple stressors early in life on a key fitness component are cumulative. We considered six environmental and social factors as known or potential ELAs to explore their effects on the survival of 242 individuals from 17 groups studied over 25 years at Kirindy Forest.

First, because of the crucial dependence of mammalian young on lactation, maternal loss has been identified as a major predictor of infant survival in all studies that included this factor. Because early maternal loss has also been identified as a major source of infant mortality in another sifaka population (Zipple et al. 2021), we expected it to have a similar effect in our study population. Second, maternal age is known to positively affect the probability of giving birth (Kappeler and Fichtel 2024), making it a potential proxy of maternal condition. Infants born to younger mothers may therefore be disadvantaged. Third, survival is compromised in larger groups (Kappeler and Fichtel 2024), suggesting that large group size at birth is a potential ELA for sifakas. Fourth, extreme climatic conditions, such as droughts, cyclones or high daily temperatures, may create physiological challenges for thermoregulation and food availability (Campos et al. 2017; Khera et al. 2025; Veilleux et al. 2024). We therefore examined the effects of rainfall and maximum temperature in early life in the highly seasonal habitat of these sifakas on subsequent survival. Fifth, in some Old World monkeys the presence of a younger sibling was shown to act as an ELA by diverting maternal resources from the focal infant (Tung et al. 2016; Patterson et al. 2024), but the presence of an older sibling in the year before an infant's birth may indicate competition for maternal care or poorer maternal condition at birth in species that follow an income breeding strategy, such as these sifakas (Lewis and Kappeler 2005). We therefore examined the effect of having an older sibling that was born in the year preceding the focal infant's birth and survived for at least 18 months, thereby ensuring temporal overlap between siblings, on the focal infant's survival. If a mother gave birth to an infant that survived at least 18 months, she likely invested substantial energy in rearing that offspring and may therefore have been in poorer condition compared to years in which she did not give birth or in which the older infant died earlier (before 18 months). Finally, we also acknowledge the fact that illness or injury in juveniles may act as important ELAs, but we have no systematic data on these events.

Because it is not possible to predict whether these effects act independently or cumulatively, we ran separate models to compare these possibilities; also, to determine the relative importance of individual stressors (see also Gicquel et al. 2022; Morrison et al. 2023; Ortiz‐Ross and Blumstein 2024). We examined these effects on survival in different life stages: early infant survival up to 15 months, survival from 15 months until adulthood, and female longevity in adulthood.

Note that, in contrast to a few previous studies (e.g., Douhard et al. 2014; Gicquel et al. 2022; Gonzalez et al. 2023; Morrison et al. 2023; Ortiz‐Ross and Blumstein 2024; Patterson et al. 2024), who conducted sex‐specific survival analyses at different life stages, high infant mortality before sifaka infants could be sexed precluded such an analysis in the present study, but sex‐specific effects were included in the survival to adulthood analysis.

## 2 Methods

### 2.1 Study System

This study was conducted on a population of Verreaux's sifakas at Kirindy Forest, Western Madagascar. Since 1994, all animals within a ca. 130 ha study area have been individually marked with unique nylon or radio collars, either when they were about 8 months old or when they immigrated into one of the study groups (Rudolph et al. 2019). Over the decades, study groups went extinct or were added to the study population as the study area expanded in size. Our analyses are based on demographic data collected during multiple weekly censuses of all 17 groups that were part of the study population at some point. Between 1995 and 2023 a total of 242 single infants were born into these groups (Table 1). An individual was pronounced dead when either a successful predation event was observed, its remains were found or when the individual was less than 8 months old at the time of disappearance, and hence, barely weaned (Malalaharivony et al. 2021). Older individuals who disappeared from their group were classified as “unknown,” as we could not unequivocally establish whether they had emigrated from the study area or died.

**TABLE 1:** Number and fate of individuals born in the population surviving different age classes.

Fate of individuals (sex of individuals)	*N* individuals	*N* individuals alive	*N* individuals dead/disappeared
Infants surviving to 15 months	242	103 (43 F, 60 M)	139 (26 F, 39 M, 74 U)
Infants surviving to 15 months with older sibling	216	92 (35 F, 57 M)	124 (25 F, 36 M, 63 U)
Infants 15 months—5 years	92	62 (20 F, 42 M)	30 (15 F, 15 M)
Females surviving > 5 years	20	7	13

*Note:* In brackets number of individuals by sex (F, female, M, male, U, unknown).

To compare short‐term consequences for infant survival with long‐term consequences for later survival at different life stages, we estimated the effect of ELAs at three different milestones: (i) ‘infant survival’ during the first 15 months of life, (ii) “survival‐to‐adulthood” for individuals from 15 months until they reached sexual maturity at an age of 5 years, and (iii) “longevity” in adulthood for females who died or disappeared at an age > 5 years.

We considered only females for the ‘longevity’‐model because males disperse from their natal groups (Leimberger and Lewis 2017), making it challenging to differentiate between dispersal and death when they disappear. We used the following classifications for dead individuals when exploring the effects of ELAs on these three estimates. First, the majority of infants (57%, *N* = 139; Table 1) disappeared within the first 15 months of life. We considered all of them to have died because they are fully dependent on maternal care in the first couple of months (Malalaharivony et al. 2021). Second, the data set for ‘survival‐to‐adulthood’ comprised *N* = 30 deaths (Table 1). Since females disperse only very rarely from their natal group (Kappeler and Fichtel 2012), we considered all disappeared females younger than 5 years as dead. Males were considered dead when they disappeared before the age of 4.59 years, which represents the first quantile of the age distribution of successful emigrations. We additionally fitted a model using the first decile of the age distribution of successful emigrations to assess the robustness of our results (see Table S1). Third, to assess the effects of ELAs on “female longevity” in adulthood, we considered only females.

### 2.2 Definition of Early Life Adversities

We defined adversity as any environmental and social condition that can have a detrimental influence on fitness. We included maternal age at birth, group size at birth, cumulative rainfall during the first 15 months of life (as proxy for food availability), the mean of the monthly maximum temperatures during the first 15 months of life, early maternal loss, and the presence of an older sibling as potential ELAs in the present analyses. We explored two thresholds for determining when a continuous variable is adverse. Following Ortiz‐Ross and Blumstein (2024), we considered a given value for a continuous variable as “moderately adverse” when it fell above the upper (third) quantile of the distribution of the respective variable (Figure S1a–e). We assigned these values a value of 1 and all values below the upper quantile a value of 0. Additionally, we considered a value of a continuous variable as “acutely adverse” when it fell above the upper decile of the distribution. We assigned these values a value of 1 and all values below the upper decile a value of 0 (Figure S1a–e). We considered binary adversities if the mother died within the first 15 months of life and assigned a value of 1 (yes) or 0 (no) accordingly. Although we assumed that the presence of an older sibling may have a negative effect on infant survival, the model including all predictors revealed that the presence of an older sibling has a positive effect on survival. Therefore, we assigned a value of 1 if there was no older sibling present and a value of 0 if there was an older sibling present. To estimate cumulative ELAs (cELAs), we created two indices: “moderate cELAs” included the sum of binary adversities (yes = 1, no = 0) plus the sum of the assigned values above the upper quantile. “Acute cELAs” included the sum of binary adversities (yes = 1, no = 0) plus the sum of the assigned values above the upper decile.

### 2.3 Analyses

All analyses were conducted using R (R Core Team 2024). To estimate the effects of ELAs on infant survival, we estimated mixed‐effects Cox Proportional Hazard models using the package “survival” (Therneau 2024). As response, we included age of individual survival for the respective lifespan considered in the given model. As predictor variables, we included whether the focal individual's mother died within its first 15 months of life (yes, no), maternal age at birth, the presence of an older sibling from the mother's previous birth (yes, no) in the year preceding the focal infant's birth, group size at the focal infant's birth and group size in the month of its death; the latter two of which correlated positively with each other (Pearson's correlation: *r* = −0.56, *N* = 242, *p* < 0.001). We included group size in the month of death in the models because previous research showed that group size has an impact on survival (Kappeler and Fichtel 2024). As environmental factors, we included cumulative rainfall and the mean of the 15 monthly maximum temperatures (*T*_max_) within the first 15 months of life, which correlated negatively with each other (Pearson's correlation: *r* = −0.47, *N* = 242, *p* < 0.001). Mother's identity was included as frailty term (see SEM Table S2 for an overview of model structures).

At the beginning of the study (in 1994), we had no information on the potential existence of older siblings. The same held true for offspring in new study groups that were successively added to the study population. As a result, this information is missing for a total of 26 focal infants. We first fitted the model without this predictor on the full data set (*N* = 242 infants), and then on the subset of the data (*N* = 216, Table 1) including this predictor. As the presence of an older sibling was found to have a significant effect on survival, we estimated the following models on the reduced data set. Next, we estimated two additional models by only including the cELA value for moderate and acute adversities and group size at death. No infant experienced all six adversities and nine infants experienced five adversities (Table 2). To balance the sample size for each category of number of adversities experienced, we grouped infants who experienced five adversities in the category of infants who experienced four or more adversities. Few infants experienced no adversities; most infants experienced one to two moderate ELAs, and only few experienced three or more than four moderate ELAs (Table 2). Regarding acute ELAs, much more individuals experienced no adversities, or only one to two, and a few individuals experienced three or more.

**TABLE 2:** Number of infants experiencing different numbers of moderate and acute ELAs.

Number of adversities	0	1	2	3	4	5	6
*N* individuals < 15 months experiencing:							
Moderate ELAs	2	52	73	53	27	9	0
Acute ELAs	33	85	69	25	3	1	0
*N* individuals > 15 months and 5 years experiencing:							
Moderate ELAs	1	28	30	24	9		
Acute ELAs	17	43	25	6	1		
*N* females > 5 years experiencing:							
Moderate ELAs	0	6	9	3	2		
Acute ELAs	4	9	5	2	0		

To estimate the effect of ELAs on “survival‐to‐adulthood,” we fitted three models including survival between 15 months and 5 years as response variable, all single predictor variables mentioned above, as well as the cELA value for moderate and acute adversities (Table S2). Since sample size decreased in the “female longevity in adulthood” model, with only 20 females surviving beyond 5 years, we restricted the set predictors to those that significantly influenced survival in the ‘survival‐to‐adulthood’ model, namely rainfall in the first 15 months of life, group size in the month of death, and mother's age, in the single predictors model (Table S2). In addition, we fitted two models including the cELA value for moderate and acute adversities.

Continuous variables were z‐transformed. Model assumptions of proportional hazards were checked by using the cox.ph function of the “survival” package (Therneau 2024). Most models met the proportional hazards assumption, except for the predictor of moderate cELAS in the “early infant survival” model and sex for all in the ‘survival‐to‐adulthood’ models. In these cases, we estimated models with a time‐dependent effect for the respective predictor. Model comparisons were conducted by calculating the AICc values based on the number of events in the respective Cox proportional hazard model. In addition, we checked for collinearity by determining Variance Inflation Factors (VIF) for a standard linear model without random effects using the package “car” (version 3.0.11; Fox and Weisberg 2019). Although rainfall and mean maximum temperature during the first 15 months of life as well as birth group size and group size in the month of death were correlated with each other, respectively (see above), variance inflation factors indicated no problematic collinearity (maximum VIF = 1.66).

## 3 Results

### 3.1 Infant Survival

**FIGURE 1:**
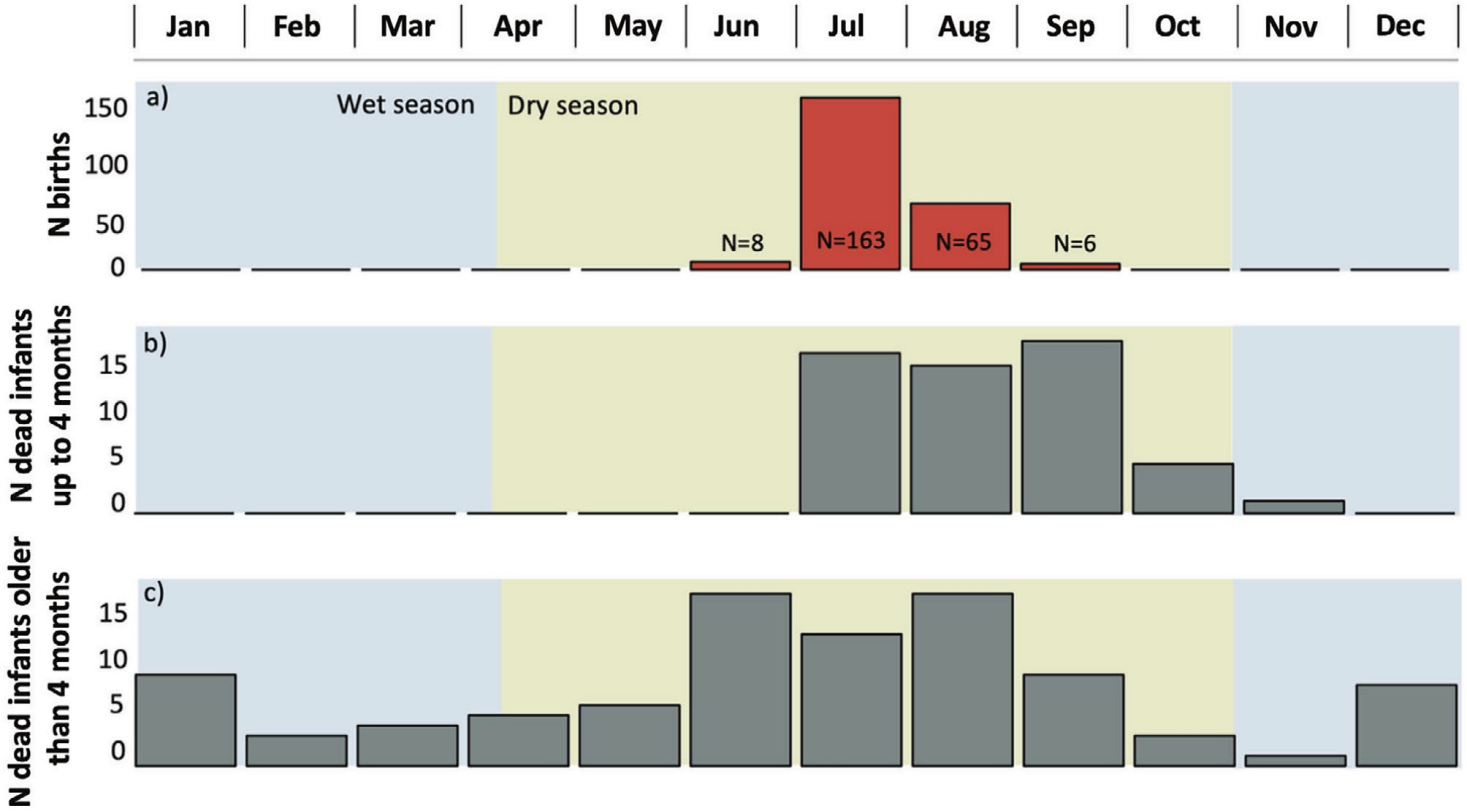
Births and deaths of Verreaux's sifaka at Kirindy Forest. (a) Number of births in a given calendar month. (b) Number of infants that died in a given month within their first 4 months of life. (c) Number of infants that died in a given month between the age of about 4–15 months of life. Light blue background indicates the wet season and the light green background the dry season in (a–c).

**FIGURE 2:**
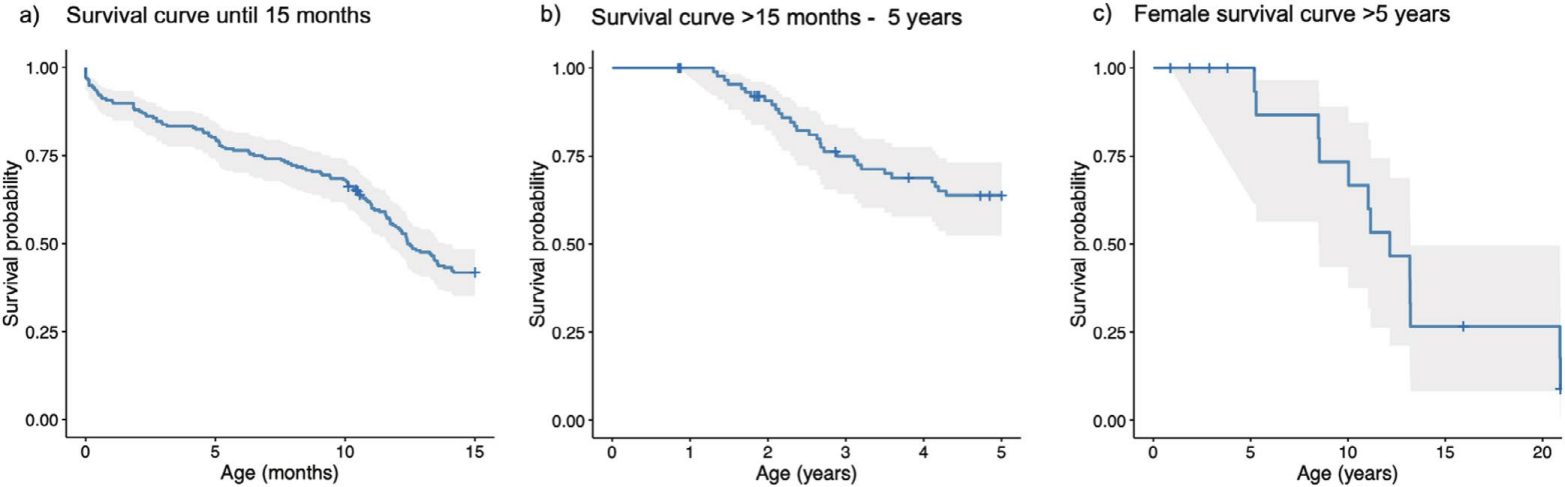
Cumulative survival probability of Verreaux's sifaka until (a) 15 months of age, (b) > 15 months and 5 years of age, and (c) female lifespan > 5 years. The blue line indicates the median and the gray polygons represent the 95% CIs.

The subsequent analyses are based on the fates of 242 infants born to 42 unique mothers residing in 17 study groups between 1995 and 2023. Reproduction was highly seasonal: infants were born between June and September, with the majority of them (*N* = 163) being born in July (Figure 1a). The majority of infants (57%, *N* = 139) died within the first 15 months of life, after which mortality rates were much lower (Figures 1b,c and 2a).

**FIGURE 3:**
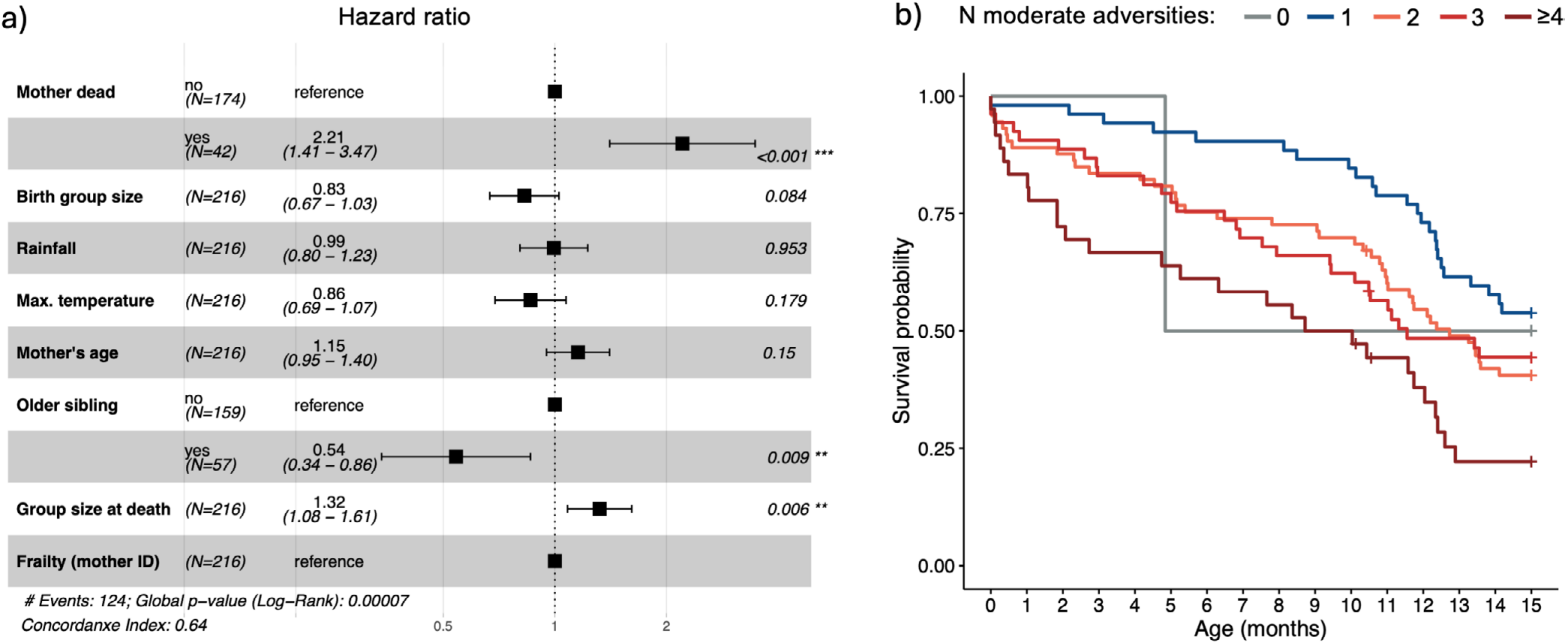
Results of the Cox proportional hazards model estimating infant survival during the first 15 months of life. (a) Forest plot of the model including all predictors and (b) survival curve depicting the moderate cumulative adversities index.

The Cox proportional hazards model including the full data set (*N* = 242 infants) revealed that the death of the mother and large group size in the month of death decreased the odds of infant survival significantly (likelihood ratio test: 18.91, df = 6, *p* = 0.004, Figure 3a, Table S3). Variation in rainfall, *T*_max_, birth group size, and mother's age did not influence the odds of infant survival. In the model with the reduced data set (*N* = 216 infants), we also found that the death of the mother and large group size in the month of death decreased the odds of infant survival (likelihood ratio test: 30.53, df = 7, *p* < 0.001, Table 3a, Figure 2a). The presence of an older sibling, however, increased the odds of infant survival, whereas rainfall, *T*_max_, birth group size, and mother's age had no influence in this model. Hence, infants whose mother died within the first 15 months of their life and those living in larger groups exhibited reduced survival, whereas infants having an older sibling that was born in the preceding year had higher survival.

**TABLE 3:** Outcome of the mixed‐effects Cox proportional hazard models estimating the influence of ELAs and cELas on infant survival to an age of 15 months. (a) Single predictors model including all categorical and continuous variables, (b) Moderate, and (c) Acute cumulative adversities indices.

Models “Early infant survival”	Term	HR	95% CI	*p*
(a) Single predictors model	Mother dead (yes, no)	2.08	1.41–3.47	**< 0.001**
	Birth group size	0.83	0.67–1.3	0.084
	Rainfall	0.99	0.80–1.24	0.950
	*T*_max_	0.86	0.69–1.07	0.180
	Mother's age	1.15	0.95–1.40	0.150
	Presence of older sibling	0.54	0.34–0.86	**0.009**
	Group size in the month of death	1.321	1.08–1.61	**0.006**
	Frailty (Mother ID)			0.920
(b) Cumulative moderate adversities	Moderate cELA index	1.91	1.26–2.88	**0.002**
	tt(Moderate cELA)	0.84	0.68–1.02	0.082
	Group size in the month of death	1.27	1.07–1.50	**0.007**
	Frailty (Mother ID)			0.920
(c) Cumulative acute adversities	Acute cELA index	1.38	1.33–1.67	**0.001**
	Group size in the month of death	1.16	0.98–1.37	0.093
	Frailty (Mother ID)			0.920

*Note:* Bold values indicates statistically significant.

Abbreviations: HR, hazard ratio; CI, confidence interval; p, exact probability.

Moderate cumulative adversities (cELAs) decreased the odds of infant survival, with infants experiencing more early‐life adversities exhibiting reduced survival (Table 3b; Figure 3b; cumulative moderate adversities: likelihood ratio test: 20.66, df = 2, *p* < 0.001). There was suggestive evidence of a time‐varying effect, indicating that moderate cELAs had a stronger effect at younger ages, which declined by age. In addition, group size in the month of death increased the odds of survival for infants living in larger groups (Table 3b). Acute cELAs also decreased the odds of survival, with infants experiencing more early‐life adversities exhibiting reduced survival (Table 3c; likelihood ratio test: 14.93, df = 2, *p* < 0.001). However, group size in the month of death had only a negative effect by trend. Of the three models, the single predictors model provided a better fit than the cumulative adversities models, with the acute cELA model providing a better fit than the moderate cELA model (AICc: single predictors model = 1223.09, moderate cELAs = 1229.39, acute cELAs = 1227.83).

### 3.2 Survival‐to‐Adulthood

The single predictors model estimating the influence of ELAs on “survival‐to‐adulthood” (*N* = 92) revealed that juveniles of older mothers and those living in large groups in the month of death experienced lower survival (likelihood ratio test: 82.67, df = 25.73, *p* < 0.001, Table 4a). Sex influenced the odds of survival, with males having a higher risk of death at an early age (> 15 months), but this effect diminished around an age of about 2 years (Figure S2). In contrast, rainfall increased the odds of survival to adulthood. Juveniles who experienced higher rainfall in the first 15 months of life had higher chances to survive until adulthood. Birth group size and *T*_max_ had no effect on survival. Both moderate and acute cELAs did not influence the odds of survival (Table 4b,c; moderate cELAs: likelihood ratio test: 41.86, df = 16.59, *p* < 0.001; acute cELAs: likelihood ratio test: 39.57, df = 16.32, *p* = 0.001). In both cELA models, juveniles living in larger groups in the months of death experienced a higher mortality risk. Sex influenced the odds of survival in these models only by trend. Here, the single predictors model provided a better fit than the cumulative ELA models, with the moderate cELA model providing a better fit than the acute cELA model (AICc: single predictors model = 231.11, moderate cELAs = 243.59, acute cELAs = 247.99).

**TABLE 4:** Outcome of the mixed‐effects Cox proportional hazard models estimating the influence of ELAs and cELAs of juvenile (> 15 months) survival until adulthood (5 years). (a) Model including single predictors, (b) moderate, and (c) acute cumulative adversities indices.

Models “Survival‐to adulthood”	Term	HR	95% CI	*p*
(a) Single predictors model	Mother dead (yes, no)	0.60	0.09–4.16	0.607
	Birth group size	1.48	0.79–2.78	0.224
	Rainfall	0.32	0.12–0.87	**0.025**
	*T*_max_	0.76	0.32–1.79	0.527
	Mother's age	2.47	1.19–5.13	**0.015**
	Presence of older sibling	1.07	0.36–3.17	0.906
	Group size in the month of death	3.51	1.78–6.94	**< 0.001**
	Sex	353.68	2.62–47736.98	**0.019**
	tt(sex)^a^	0.007	0–0.31	**0.011**
	Frailty (Mother ID)			**< 0.001**
(b) Moderate cumulative adversities	Moderate cELA index	1.35	0.85–2.13	0.200
	Group size in the month of death	1.66	1.05–2.62	**0.031**
	Sex	68.32	0.91–5112.95	0.055
	tt(sex)^a^	0.02	0.0008–0.76	**0.034**
	Frailty (Mother ID)			**0.039**
(c) Acute cumulative adversities	Acute cELA index	1.02	0.62–1.69	0.930
	Group size in the month of death	1.59	1.03–2.47	**0.038**
	Sex	51.54	0.71–3719.77	0.071
	tt(sex)^a^	0.03	0.001–0.87	0.041
	Frailty (Mother ID)			**0.045**

*Note:* Bold values indicates statistically significant.

Abbreviations: HR, hazard ratio; CI, confidence interval; p, exact probability.

^a^ tt() denotes the time‐dependent component of sex, included to account for non‐proportional hazards.

### 3.3 Female Longevity

Since only 20 mothers survived for more than 5 years, we included only the combination of predictors that had a significant influence on survival in the ‘survival‐to‐adulthood’ model, that is, rainfall in the first 15 months of life, group size in the month of death, and mother's age, in the single predictors model (Table 5). Two predictors were selected for each model, resulting in three models covering all potential combinations of predictors. The single predictors model estimating the influence of ELAs on female longevity in adulthood including rainfall in the first 15 months of life and group size in the month of death provided the best fit (Table S4). Both predictors had a significant influence on female longevity, with females experiencing as infants (in the first 15 months of life) higher rainfall exhibiting higher survival, whereas females living in larger groups in the month of death exhibited lower survival (AICc:48.17; likelihood ratio test: 10.06, df = 2, *p* = 0.007, Table 5a). Neither the moderate nor the adverse cumulative adversities model influenced the odds of survival (Table 5b,c; moderate cELAs: likelihood ratio test: 6.56, df = 2, *p* = 0.040; acute cELAs: likelihood ratio test: 5.89, df = 2, *p* = 0.050). In both models, group size in the month of death decreased the odds of survival, with females living in larger groups experiencing lower survival. The model including the single predictors provided a better fit than the cumulative ELA models (AICc: single predictors model = 48.17, moderate cELAs = 51.66, acute cELAs = 52.34).

**TABLE 5:** Outcome of the mixed‐effects Cox proportional hazards models estimating the influence of ELAs and cELAs on female longevity: (a) Model including the two predictors, (b) moderate, and (c) acute cumulative adversities indices.

Models for ‘female longevity’	Term	HR	95% CI	*p*
(a) Single predictors model	Rainfall	0.32	0.11–0.93	**0.036**
	Group size in the month of death	3.74	1.54–9.10	**0.004**
	Frailty (Mother ID)			0.960
(b) Moderate cumulative adversities	Moderate cELA index	0.52	0.17–1.56	0.240
	Group size in the month of death	2.13	1.07–4.24	**0.032**
	Frailty (Mother ID)			0.960
(c) Acute cumulative adversities	Moderate cELA index	1.41	0.64–3.11	0.400
	Group size in the month of death	2.17	1.08–4.37	**0.030**
	Frailty (Mother ID)			0.960

*Note:* Bold values indicates statistically significant.

Abbreviations: HR, hazard ratio; CI, confidence interval; p, exact probability.

## 4 Discussion

We studied the effects of early‐life adversities on survival during three stages across the lifetime of Verraux's sifakas. First, our analyses revealed that maternal loss and group size at time of death had negative effects on infant survival during the first 15 months of life, whereas the presence of an older sibling had an unexpected positive effect. As in all previous studies, cumulative ELAs had negative effects on early infant survival, but here the single predictor model provided a better fit, suggesting that a mother's death has the strongest effect. Second, subsequent survival from 15 months to adulthood at 5 years was negatively affected by mother's age at birth and group size at the time of death, whereas rainfall in the first months of life had a positive effect on surviving this stage, but we detected no effects of cELAs. Young males were less likely to survive this stage than females. Third, the longevity of adult females beyond 5 years of age was positively affected by rainfall experienced early in life and decreased as a function of group size at the time of death, whereas cELAs had no effect. Hence, maternal loss appears to be the most important predictor of infant mortality. The effects of other factors can be compensated later on, except for the negative effects of living in a large group (Kappeler and Fichtel 2024). We discuss these main findings in the context of the design and results of previous studies below.

### 4.1 Choice of Models and Indices

Our study confirmed some of our predictions as well as observations in other taxa, but it also revealed some deviant patterns and yielded unexpected insights. First, cumulative ELAs had, independent of the particular sample details, a negative impact on sifaka survival, but only during the first 15 months of life. Our study therefore adds to previous analyses in wild large mammals (see above) that found support for the cumulative stress hypothesis, postulating that multiple insults in early life have cumulative negative effects on survival (Stewart 2006). Considering the effects of multiple factors in models is therefore a logical choice adopted by virtually all recent studies of wild vertebrates. However, because individual adversities may exhibit complex interactions, additive, antagonistic or synergistic effects (Orr et al. 2020; Pirotta et al. 2022), using a cumulative index may represent a superior analytical strategy. Large sample sizes allow to use and compare all three approaches empirically (spotted hyenas: Gicquel et al. 2022), but this has not been possible in other studies, even though they spanned several decades. Because this spotted hyena study revealed that multivariate models outperformed most cumulative and single‐effect models, future studies with large sample sizes will have to clarify the relationship between multivariate and cumulative effects models to guide the way for future analyses.

The choice of a meaningful cumulative index is not straightforward, however. As first shown for wild savannah baboons (Tung et al. 2016), a simple index based on the summed number of ELAs experienced was sufficient and predicted female survival better than an individual effects model. In a yellow‐bellied marmot study, however, the full model offered a better prediction of pup survival than models with cumulative indices, whereas most of the full models did not predict adult survival, while the cumulative adversity indices did (Ortiz‐Ross and Blumstein 2024). The yellow‐bellied marmot study introduced cumulative adversity indices with nuanced estimates of the insults (moderate vs. acute) and also used one with summed standardized scores. In a study of red squirrels (*Tamiascurus hudsonicus*), a weighted index integrated the sum of ELAs and their respective effect sizes (Petrullo et al. 2024), thereby offering a more fine‐grained measure. A study on mountain gorillas also used a numerical index of cumulative ELAS, but also employed a dynamic index, for which putative sources of ELA were updated at the point in time at which an individual experienced an additional insult (Morrison et al. 2023). These individual and the two cumulative effect models had similar levels of goodness of fit, suggesting that dynamic cumulative indices offer no superior resolution, but more applications of such a time‐sensitive measure in other studies would be welcome.

We found that the single predictors model provided a better fit than the cumulative models for infant sifaka survival. The acute cELA model predicted the sifaka data slightly better than moderate cELA model, suggesting that extreme values of the stressors may have a stronger impact of infant survival. Combining continuous and categorical measures into a single composite index may introduce an additional source of variation across studies, however (see Ortiz‐Ross and Blumstein 2024). Weighing individual adversities across measures may be a meaningful option (e.g., Petrullo et al. 2024), but this requires the availability of detailed data on the fitness effects of each measure. A better understanding of the pros and cons of using different indices should be an important goal of future studies because they can also provide insights into the mechanistic underpinnings of developmental trade‐offs and their consequences (Eyck et al. 2019).

### 4.2 Time Scales

Second, a key question about ELAs concerns their timing. The period of time over which effects of ELAs can be discerned is interesting because it may inform efforts to illuminate the types of possible responses to developmental stressors and the underlying mechanism (Eyck et al. 2019; Lea and Rosenbaum 2020; Lu et al. 2019). Because the conceptual focus in these studies is on adverse effects taking place during the pre‐ and or early postnatal period, it is obvious for studies to examine immediate effects on infant survival. Both, the absolute length of the time windows during which stressors can impact subsequent development and when and how long their effects are discernible have varied in previous studies, however. For example, yellow‐bellied marmots reach sexual maturity at age 2, and Ortiz‐Ross and Blumstein (2024) therefore considered the first 2 years (analyzed separately and in combination) as the early life period, which covers most of their average lifespan (3.8 years). In the other temperate rodent study (red squirrels), the end of the first winter (@ > 200 days) provided the critical milestone for exploring effects of ELAs on juvenile survival (Petrullo et al. 2024). One study on female spotted hyenas defined early life as the first 6 months of life and examined variation in cub growth rates and juvenile survival, reporting that cELAs affected survival to adulthood (at age 2 years) and lifetime reproductive success (Gicquel et al. 2022); in another spotted hyena study, another set of cELAs negatively affected adult lifespan (Strauss et al. 2020). In savannah baboons, Tung et al. (2016) considered time until reproductive maturity (i.e., 4 years) as early life and examined the effects of ELAs on survival throughout the lifespan. In wild gorillas, the critical period of early life extended up to 6 years of age (Morrison et al. 2023). They suffered a massive (> 26‐fold) reduction in early survival if they experienced the cumulative effects of three ELAs, but the surviving gorillas did not suffer greater mortality as adults. In rhesus macaques, the definition of early life also relied on sexual maturity (3 years) as a defining milestone, and the analyses examined the effects on infant and adult survival separately (Gonzalez et al. 2023). We divided the age at sexual maturity into two stages, where the 15 months mid‐milestone was chosen based on the observed mortality pattern. Thus, independent of the absolute length of the chosen time window for the sensitive period and the visibility of the response, most wild mammals suffer fitness costs from cELAs across the lifespan. It would seem useful to frame the chosen time window in relation to key developmental milestones to facilitate future comparisons, however.

### 4.3 Stressors

Third, recent studies of multiple ELAs focused on different potential stressors, justifying their choice through direct links to a given study species' life history via a known fitness link. Given this heterogeneity across studies, it may be helpful for future studies to group the specific insults into categories, such as “social,” “demographic,” and “environmental,” for example, so as to facilitate comparisons across studies and species, while acknowledging that species‐specific ecological and social contexts may necessitate tailored adversity measures and that components of these categories may interact with each other. Broader discussion of such a classification approach is clearly warranted.

Of the various factors that could be considered to represent social stressors, maternal loss appears to represent the most powerful adversity that typically results in early infant death—also in this study. Before weaning, the mechanism underlying infant death in mammals is evident, but maternal social support later on in life may be important as well (see Tung et al. 2016); especially for the philopatric sex (Stanton et al. 2020). Importantly, if much of the maternal loss effect comes from mothers dying before their infants, it is more akin to a sudden response to a major environmental insult, not a long‐term early life effect. When mothers died relative to the deaths of their infants is therefore crucial for the interpretation of this effect. In our study, 153 infants died before the mother died, 33 infants died at about the same time—quite likely as a result of simultaneous predation—and 62 infants died after the mother did, but in only 2 cases were the infants still dependent. Thus, in this study maternal loss had more often long‐term, rather than immediate effects on infant survival. More recently, the positive effects of father‐offspring relationships on female survival have been recognized in baboons, even though paternal care is subtle or indirect (Jansen et al. 2025). Experiments with captive rhesus macaques also revealed that the lack of a stable, secure attachment relationship with the mother leads to detrimental health consequences, even if maternal loss is compensated by a surrogate mother (Conti et al. 2012). ELAs, such as maternal loss, and social relationships were found to have independent effects on survival in wild baboons (Lange et al. 2023), indicating that social adversities can extend beyond the mother‐infant bond.

Presence of a close‐in‐age sibling or litter size has been included in several studies as another potential social adversity. The logic here is that either one of two closely‐born siblings or the members of larger litters may suffer from reduced parental investment and competition from the other sibling for resources and parental attention (Delaunay et al. 2024; Hudson and Trillmich 2008). In rhesus monkeys, presence of a younger sibling had no effect on male and female infant survival (Gonzalez et al. 2023; Patterson et al. 2024), whereas presence of a younger sibling nearly doubled a female's mortality hazard at every age in savannah baboons (Tung et al. 2016). In contrast, the present study found that the presence of an older sibling was associated with higher infant survival in sifakas, suggesting that maternal condition rather than sibling competition influences infant survival in this species. Because the presence of a younger sibling reduced adult mortality risk in gorillas (Morrison et al. 2023), effects of the presence of an older or younger sibling are not consistent across species and deserve more study.

Maternal dominance rank is another potential social factor to consider because higher rank tends to be positively associated with resource access, and thus the ability to invest more into offspring. Surprisingly, in spotted hyenas maternal rank had no effect on female cub growth rates and survival to adulthood (Gicquel et al. 2022), and in savannah baboons it did not impact offspring survival (Tung et al. 2016). In sifakas, about half of the groups contain only a single adult female, preventing us from including maternal rank in a meaningful way in our analyses.

Maternal age at birth is one of the demographic factors often included as potential adversities because young mothers may be less experienced and old mothers may suffer from compromised condition. In spotted hyenas, maternal age was the strongest single predictor of survival to adulthood and female lifetime reproductive success (Gicquel et al. 2022), and in rhesus macaques, infants of a primiparous mother also experienced an increased risk of death (Gonzalez et al. 2023). In sifakas, having an older mother decreased the odds of juvenile survival, contributing an additional example of this effect, but these lemurs exhibit no reproductive senescence (Kappeler, Pethig, et al. 2022). Thus, maternal age appears to be a significant predictor of infant and/or juvenile survival, independent of other factors, and across vertebrates and invertebrates (Ivimey‐Cook and Moorad 2020), and should therefore be included in more future studies of ELAs.

Group size and population density represent a set of demographic factors that may impact infant survival. On the one hand, resource competition is presumably intensified in larger groups and in populations at higher densities, hampering energy access by mothers and infants during early development. In bighorn sheep, lambs born at high population densities suffered indeed from overall reduced survival and reproductive success as adults (Douhard et al. 2019; Pigeon et al. 2017). On the other hand, survival should be enhanced in larger groups in species in which predation is the main source of mortality. In spotted hyenas, the number of lactating females, an operational measure of group size, had a strong dampening effect on cub growth and survival to adulthood (Gicquel et al. 2022), reflecting heightened competition among females within clans. In rhesus macaques, minimal increases in the number of adult females had measurable negative consequences for infant survival, and this despite daily provisioning with *ad libitum* monkey chow (Gonzalez et al. 2023). In wild savannah baboons, in contrast, variation in group size had no effect on longevity (Tung et al. 2016).

In sifakas, growing up and living in a larger group negatively impacted survival at all ages. A previous study examining behavioral, ecological and physiological correlates of group size variation in sifakas found no evidence for effects of group size on any of these measures (Rudolph et al. 2019). Large group size does not appear to offer any of the expected anti‐predator benefits, but juveniles are less vigilant (Schumacher et al. 2025) so that the high observed juvenile mortality may be due in part to their smaller size and limited antipredator abilities. Increased female competition for resources in larger groups may also play a role in this case because female reproductive rates are negatively affected by the number of resident adult females (Kappeler and Fichtel 2024), but the negative effects of living in large groups on survival of sifakas remain obscure.

Many studies investigated down‐stream fitness consequences of climatic conditions or events. In temperate species, like the yellow‐bellied marmots, the length of the winter or summer rainfall impacted food availability for mothers and their developing offspring. In this study, summer droughts actually improved pup survival, but the mechanisms underlying this effect remain obscure (Ortiz‐Ross and Blumstein 2024). In tropical savannah baboons, experiencing a drought in the first year of life had no effect on longevity (Tung et al. 2016). In sympatric African elephants, droughts increased mortality among the youngest and oldest individuals, but they had no impact on later female reproduction (Lee et al. 2022). In contrast, rainfall was the best single factor explaining female adult survival in spotted hyenas living in a similar habitat (Gicquel et al. 2022). In bighorn sheep, lamb survival was compromised by precipitation at birth (Pigeon et al. 2017). In the present study, rainfall during the first 15 months of life had a positive effect on survival to adulthood and female longevity. However, maximum temperatures had no effect on sifaka survival at any age, whereas sympatric, but much smaller gray mouse lemurs ( *Microcebus murinus* ) responded to increasing temperatures and reduced rainfall over the last decades with lower survival (Ozgul et al. 2023). In rhesus macaques, adult male, but not female survival was compromised by hurricanes (Gonzalez et al. 2023), and several species of seabird exhibited reduced survival after winter storms (Laurenson et al. 2025). Body size and behavior may help to mediate the immediate effects of catastrophic climatic events (Walker et al. 2023). Thus, environmental stressors appear to have the most widespread effects, but their direction can vary.

Climatic factors may not only have immediate impact, but they are also associated with future food availability. It is therefore also interesting to ask whether environmental ELAs can be compensated by better conditions during later development (Eyck et al. 2019). Red squirrels experiencing multiple ELAs suffered from shorter lifespans, but cohorts experiencing a food boom in the second year of life did not (Petrullo et al. 2024). Experimental food supplementation could not replicate this effect; however, indicating that later food availability alone is insufficient for a compensation of the effects of ELAs. Ecological sources of ELAs and cELAs had no effect on early survival in a provisioned population of rhesus macaques (Gonzalez et al. 2023), suggesting that compensatory mechanisms can be activated under the right circumstances, but the available evidence does not allow an assessment of when and how such an effect occurs.

### 4.4 Conservation Implications

Finally, given that these sifakas are classified as *Critically Endangered*, understanding the relative importance of potential drivers of mortality and their cumulative effects may provide insights about implications for conservation strategies. Madagascar is particularly hard hit by recent climate change, with maximum temperatures increasing and average annual precipitation decreasing (Hannah et al. 2008; Ingram and Dawson 2005; Ozgul et al. 2023). Survival of sifakas to adulthood and female longevity were positively affected by higher rainfall during the first 15 months of life. Hence, the observed decrease in average rainfall over the last decades indicates that, as gray mouse lemurs (Ozgul et al. 2023), the survival of sifakas is impaired by climate change. A sympatric critically endangered lemur ( *Microcebus berthae* ) already went extinct locally, perhaps due to their inability to adjust to changing environmental or ecological conditions (Kappeler, Markolf, et al. 2022). The negative effects of group size for survival may be of greatest relevance for practical conservation action: with ongoing habitat destruction and illegal hunting, an increasing number of individuals may find themselves living in ever‐smaller areas, thereby leading to unnaturally high population densities. If individuals from hunted groups join intact groups, their group size would increase, and both changes would be magnified by long‐term negative effects on survival. Thus, the protection of intact sifaka habitat is a concrete conservation recommendation emerging from the present analyses.

## 5 Conclusions

Our study supported the notion established by previous studies that studying cELAs represents a meaningful analytical strategy for identifying the effects of multiple stressors on individual fitness. Our study demonstrated that social and demographic adversities, but also environmental adversities, affected survival. As in some other studies, adversities early in life had not only immediate, but also persistent negative long‐term effects on survival, identifying them as important drivers of demography and life history. We also show that some presumed stressors—in our case the presence of an older sibling—can have opposite fitness effects than previously expected, highlighting the need to justify the choice of particular potential adversities. The discussion of the results of this study also underlined the fact that the still relatively few studies dedicated to the effects of early lifetime adversities in wild mammals used variable sets of stressors, fitness proxies and analytical procedures, hampering attempts to extract broad generalizations at this stage. Last but not least, studies of the effects of early life adversities can also provide empirically derived arguments for concrete conservation action.

## Author Contributions

**Claudia Fichtel:** conceptualization (equal), data curation (lead), formal analysis (lead), project administration (equal), writing – review and editing (supporting). **Peter M. Kappeler:** conceptualization (equal), data curation (equal), formal analysis (supporting), project administration (equal), writing – original draft (lead), writing – review and editing (equal).

## Funding

This work was supported by multiple grants by the Deutsche Forschungsgemeinschaft.

## Conflicts of Interest

The authors declare no conflicts of interest.

## Supporting information


**Figure S1:** (a–e) Distribution of continuous predictors of ELAs. (a) Cumulative rainfall during the first 15 months of life, (b) Mean monthly maximum temperature during the first 15 months of life, (c) mother's age in years, (d) group size at birth, and (e) group size at death. Yellow lines indicate the 75% quantile to assign moderate ELAs and red lines indicate the upper decile to assign acute ELAs.
**Table S1:** Outcome of the mixed‐effects Cox proportional hazard models estimating the influence of ELAs and cELas of juvenile (> 15 months) survival until adulthood (5 years) using the cutoff of the 1st percentile for age at male emigration.
**Table S2:** Overview of the model structures. For each life‐stage, we estimated three models.
**Table S3:** Outcome of the mixed‐effects Cox proportional hazard model estimating the influence of ELAs on infant survival including all infants (*N* = 242).
**Table S4:** Outcome of the mixed‐effects Cox proportional hazard model estimating the combinations of two predictors on female longevity.
**Figure S2:** Cumulative survival probability of male and female Verreaux's sifaka between the age of > 15 months and 5 years (red line = females, blue line = males).

## Data Availability

All data and code supporting the results have been archived in figshare: https://figshare.com/s/12c14ee53f9c9936b277.
