# Supplementary material for: Cumulative Early‐Life Adversities Predict Short‐ and Long‐Term Survival in a Critically Endangered Lemur ( Propithecus verreauxi )
Source: Ecol Evol. 2026 Aug 14;16(8):e74194. doi: 10.1002/ece3.74194 (PMC13476699; doi:10.1002/ece3.74194)
Supplement: Supplementary file 1 [file ECE3-16-e74194-s001.docx]

Supplementary material

for

**Cumulative early-life adversities predict short- and long-term survival in a *Critically Endangered* lemur (*Propithecus verreauxi*)**

**Claudia Fichtel | Peter M. Kappeler**

Figure S1a-e: Distribution of continuous predictors of ELAs. a) Cumulative rainfall during the first 15 months of life, b) Mean monthly maximum temperature during the first 15 months of life, c) mother’s age in years, d) group size at birth, and e) group size at death. Yellow lines indicate the 75% quantile to assign moderate ELAs and red lines indicate the upper decile to assign acute ELAs.

Table S1: Outcome of the mixed-effects Cox proportional hazard models estimating the influence of ELAs and cELas of juvenile (>15 months) survival until adulthood (5 years) using the cutoff of the 1^st^ percentile for age at male emigration.

| Models  ‘Survival-to adulthood’ | Term | HR | 95%CI | P-value |
| --- | --- | --- | --- | --- |
| a) Single predictors model | Mother dead (yes, no) | 0.60 | 0.09 – 4.16 | 0.607 |
|  | Birth group size | 1.48 | 0.79 – 2.78 | 0.224 |
|  | Rainfall | 0.32 | 0.12 – 0.87 | **0.025** |
|  | T_max_ | 0.76 | 0.32 – 1.79 | 0.527 |
|  | Mother’s age | 2.47 | 1.19 – 5.13 | **0.015** |
|  | Presence of older sibling | 1.07 | 0.36 – 3.17 | 0.906 |
|  | Group size in the month of death | 3.51 | 1.78 – 6.94 | **<0.001** |
|  | Sex | 353.68 | 2.62 – 47736.98 | **0.019** |
|  | tt(sex) | 0.007 | 0 – 0.31 | **0.011** |
|  | Frailty (Mother ID) |  |  | **<0.001** |

Results were similar to the results of the model using the first quantile of the distribution for age at male migration, indicating that our findings were robust to the choice of threshold.

Table S2: Overview of the model structures. For each life-stage, we estimated three models.

| **Survival** | **Models** | **Frailty term** |
| --- | --- | --- |
| **15 months of life** | 1) mother dead (yes, no), group size in the month of birth, annual rainfall first 15 months of life, average monthly maximum temperature first 15 months of life, mother’s age, group size in the month of death | Mother ID |
|  | 2) Moderate cELA index, group size in the month of death | Mother ID |
|  | 3) Acute cELA index, group size in the month of death | Mother ID |
| **15 months to adulthood** | 1) mother dead (yes, no), group size in the month of birth, annual rainfall first 15 months of life, average monthly maximum temperature first 15 months of life, mother’s age, group size in the month of death, sex | Mother ID |
|  | 2) Moderate cELA index, group size in the month of death, sex | Mother ID |
|  | 3) Acute cELA index, group size in the month of death, sex | Mother ID |
| **Female longevity** | 1) Rainfall, group size in the month of death | Mother ID |
|  | 2) Moderate cELA index, group size in the month of death | Mother ID |
|  | 3) Acute cELA index, group size in the month of death | Mother ID |

Table S3: Outcome of the mixed-effects Cox proportional hazard model estimating the influence of ELAs on infant survival including all infants (N=242).

| Models  “early infant survival” | Term | HR | 95%CI | P-value |
| --- | --- | --- | --- | --- |
| a) Single predictors model | Mother dead (yes, no) | 1.97 | 1.30 – 3.00 | **0.001** |
|  | Birth group size | 0.93 | 0.77 – 1.12 | 0.420 |
|  | Rainfall | 1.03 | 0.84 – 1.26 | 0.780 |
|  | Max. temperature | 0.91 | 0.74 – 1.12 | 0.380 |
|  | Mother’s age | 1.16 | 0.97 – 1.40 | 0.100 |
|  | Group size at death | 1.25 | 1.04 – 1.51 | **0.017** |
|  | Frailty (Mother ID) |  |  | 0.920 |

Table S4: Outcome of the mixed-effects Cox proportional hazard model estimating the combinations of two predictors on female longevity.

|  | Term | HR | 95%CI | P-value |
| --- | --- | --- | --- | --- |
| Models  ‘female longevity’ | Mother’s age | 0.86 | 0.38 – 1.96 | 0.730 |
|  | Group size in the month of death | 2.15 | 1.07 – 4.28 | 0.030 |
|  | Frailty (Mother ID) |  |  | 0.960 |
|  | AICc= 52.94; Likelihood ratio test: 5.29, df=2, p=0.070 | | | |
|  | Mother’s age | 0.75 | 0.35 – 1.60 | 0.410 |
|  | Rainfall | 0.71 | 0.31 – 1.60 | 0.470 |
|  | Frailty (Mother ID) | 68.32 | 0.91 – 5112.95 | 0.950 |
|  | AICc= 57.33; Likelihood ratio test: 0.9, df=2, p=0.600 | | | |

Figure S2: Cumulative survival probability of male and female Verreaux’s sifaka between the age of >15 months and 5 years (red line = females, blue line = males).
